# Design and optimization of dual-motor electric tractor drive system based on driving cycles

**DOI:** 10.1371/journal.pone.0286378

**Published:** 2023-06-02

**Authors:** Junjiang Zhang, Bin Zhao, Xianghai Yan, Mengnan Liu, Liyou Xu, Chengyan Shang

**Affiliations:** 1 College of Vehicle and Traffic Engineering, Henan University of Science and Technology, Luoyang, China; 2 State Key Laboratory of Intelligent Agricultural Power Equipment, Luoyang, China; 3 Henan Province Collaborative Innovation Center for Advanced Manufacturing of Mechanical Equipment, Luoyang, China; 4 YTO Group Corporation, Luoyang, China; Southwest Jiaotong University, CHINA

## Abstract

Aiming at the unreasonable determination of the power coupling device speed ratio and the power battery capacity in the initial design stage of the dual-motor electric tractor, a dual-motor drive system is designed, and a parameter optimization method based on driving cycles (POMBDC) is proposed. By analyzing the driving characteristics requirements and actual working conditions of the tractor, the dynamic model of the dual-motor drive system under different working modes is established, and the parameters of the dual-motor, transmission and maximum service mass are designed. On this basis, based on the driving cycles and aiming at the lowest power consumption, the POMBDC is formed, this method can collaboratively optimize the power coupling device speed ratio and the power battery capacity. In order to verify the rationality of the POMBDC, the instantaneous optimization-constant speed ratio design method (IO-CSRDM), rule-optimization speed ratio design method (R-OSRDM) and rule-constant speed ratio design method (R-CSRDM) are developed as comparison methods, and simulation experiments are carried out. Under plowing conditions, the power battery capacity of the POMBDC is 3.08%, 5.71%, and 8.73% lower than those of the IO-CSRDM, R-OSRDM, and R-CSRDM, respectively. The power consumption resulting from the POMBDC is reduced by 3.11%, 5.74%, and 8.8%, compared with those of the IO-CSRDM, R-OSRDM and R-CSRDM, respectively. Under rotary tillage conditions, the power battery capacity of the POMBDC is 6%, 8.64%, and 11.11% lower than those of the IO-CSRDM, R-OSRDM, and R-CSRDM, respectively. The power consumption resulting from the POMBDC is reduced by 6.05%, 8.66%, and 11.13%, compared with those of the IO-CSRDM, R-OSRDM and R-CSRDM, respectively. The POMBDC can effectively increase the operating mileage of pure electric tractors and reduce costs.

## Introduction

In the global agricultural machinery industry, tractors are the largest category. However, traditional internal combustion engine tractors consume a lot of fossil fuels and have poor emissions, resulting in energy shortages and environmental pollution [1–3]. With the advocacy of protecting the environment and reducing energy consumption around the world, it is of great significance to study new energy-saving tractors [4–6]. Pure electric tractor is a kind of green and environment-friendly agricultural machinery, its energy comes from motor and power battery, which can realize zero emission and overcome the problems of low efficiency, high noise and high energy consumption of traditional internal combustion engine tractors [7–9]. Therefore, pure electric tractors are getting more and more attention.

Compared with traditional internal combustion engine tractors, the operating time of pure electric tractors is relatively short. As the core of the pure electric tractor, the drive system’s parameters are of great importance to improve the operating efficiency of the whole machine and ensure the operating mileage [10–15]. Many scholars have conducted relevant research on the parameter matching design of tractor drive system. Zhang et al. [16] proposed a matching design method for solar garden tractor drive system, and carried out theoretical calculations on the main parameters. The research shows that solar gardening tractors can meet the needs of gardening operations. Fang et al. [17] proposed a selection and matching method for pure electric tractors, and analyzed the operation performance of pure electric tractors after selection, The results showed that they could meet the operation requirements of different working conditions. Zhao et al. [18] proposed a design method for extended-range electric tractors, and carried out parameter design on the main power components of the drive system. Compared with traditional tractors, the economic efficiency was increased by 5.37%. Chen et al. [19] designed a tractor electric drive system based on hilly and mountainous plowing and sowing conditions. The test results show that it meets the characteristics requirements of tractors under plowing and sowing conditions. Although the above parameter matching design method can meet the tractor’s driving characteristics and driving range index requirements, there is still room for improvement in the tractor’s energy utilization efficiency.

In recent years, in order to improve the economy of tractors, researchers have begun to optimize the parameters of the tractor drive system on the basis of parameter matching design. Fu et al. [20] used the improved nondominated sorting genetic algorithm to optimize the transmission ratio of the tractor gearbox. The results showed that the economy and power of the whole machine were improved. Wu et al. [21] used Isight optimization software to optimize the transmission speed ratio with the goal of economy. The results showed that the continuous operation time was increased by 0.3–0.5 h compared with that before optimization. Chen et al. [22] designed a method for parameter matching and optimal design of the power system of a dual-motor driven electric tractor. This method uses a particle swarm optimization algorithm based on a hybrid penalty function for parameter optimization, which prolongs the continuous operation time of the electric tractor. Li et al. [23] proposed a dual-input coupling power transmission system, and optimized the power ratio of the dual motors and the characteristic parameters of the planetary gear set. After optimization, compared with the single-motor power system, the efficiency of the whole machine was improved by about 9.8%. Wen et al. [24] proposed an innovative method to design a dual-motor power coupling drive system for an electric tractor, and the simulation results showed that the maximum working mileage was increased by 16.3%. The above achievements provide many references for the research of tractor drive system, but there are relatively few studies on optimizing the power coupling device speed ratio device and the power battery capacity at the same time.

For pure electric tractors, power battery account for a relatively large proportion of the overall cost of the tractor. Too small power battery capacity cannot meet the requirements of the working area, and too large capacity will increase the cost of the tractor. In addition, a reasonable selection of the power coupling device speed ratio can effectively improve the operating efficiency of the motor. Therefore, it is very necessary to optimize the capacity of the power battery and the speed ratio of the power coupling device.

This paper takes the dual-motor electric tractor as the research object, designs the dual-motor drive system, and designs the parameters of the dual-motor, transmission, and maximum service mass. On this basis, based on the cyclic driving cycles and aiming at the lowest power consumption, a parameter optimization method based on driving cycles (POMBDC) is formed. This method can simultaneously optimize the power coupling device speed ratio and the power battery capacity.

## Configuration and modeling of dual-motor drive system

### Configuration scheme

**Fig 1:**
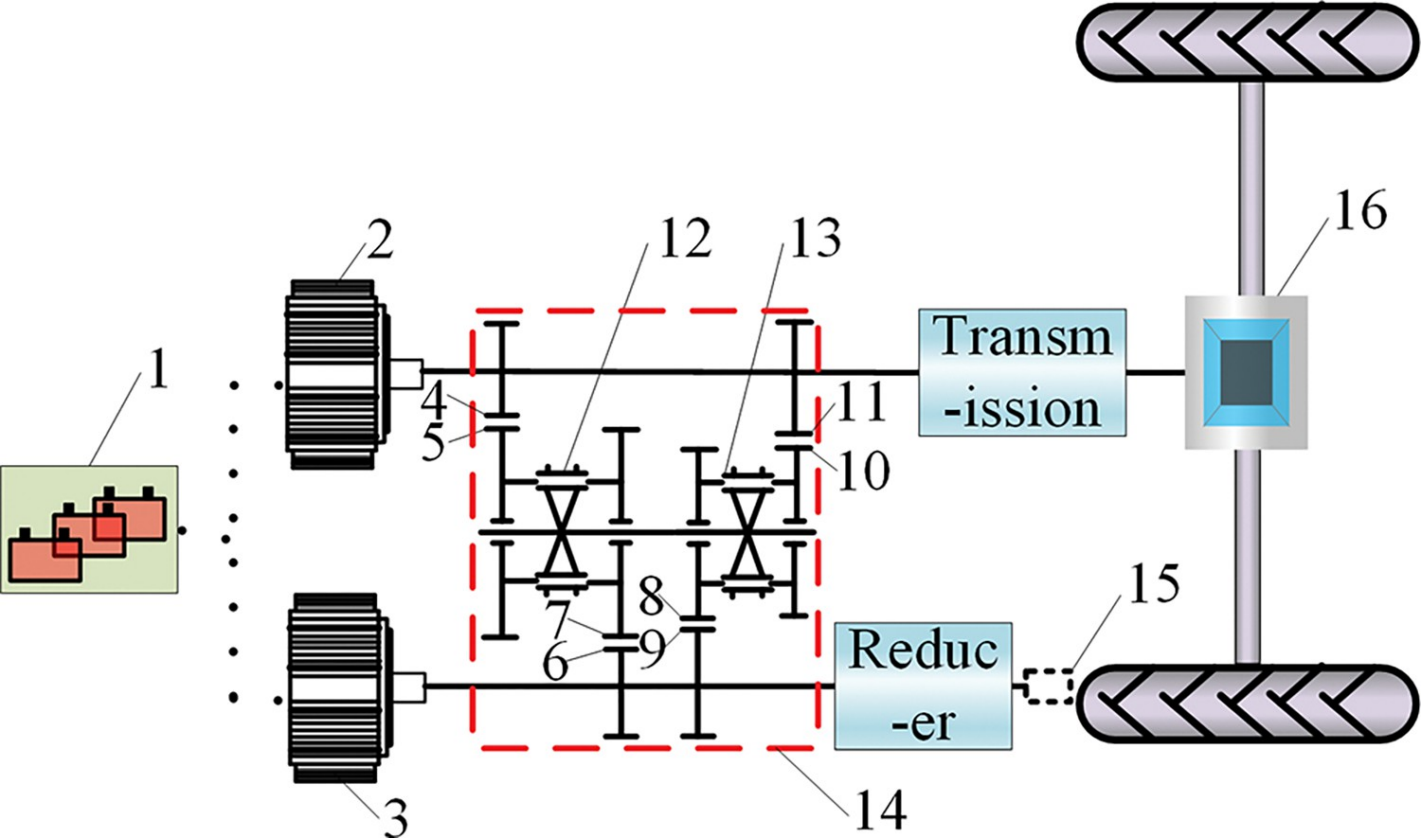
Overall configuration of dual-motor electric tractor. 1. Power battery 2. Traction motor 3.PTO motor 4~11. Transmission gear pair gear 12.Synchronizer one 13.Synchronizer two 14.Power coupling device 15. PTO 16. Central transmission device.

According to the actual operation requirements of the tractor, a dual-motor electric tractor drive system is designed, and its configuration scheme is shown in Fig 1. The main components include power battery, traction motor, power take-off (PTO) motor, power coupling device, transmission, reducer and central transmission device. Among them, the power battery supplies power to 2 electric motors, the traction motor and the PTO motor are connected to the power coupling device. One end of the power coupling is connected to the rear drive system consisting of the transmission, the central transmission device, and the rear wheels. The PTO is connected with the other end of the power coupling device through the reducer, which can provide power output for the field operation of the dual-motor electric tractor.

The motor has the characteristics of low-speed constant torque and high-speed constant power, so the dual-motor driven electric tractor can use a gearbox with fewer gears. In order to meet the motor performance requirements of the dual-motor electric tractor under different working conditions, high and low gear transmissions are used in this paper.

#### Dynamic model of dual-motor drive system

The dual-motor drive system can realize three operation modes under the driving state of the tractor: single-motor drive mode, dual-motor coupling drive mode, and dual-motor coupling rotary tillage mode.

**Fig 2:**
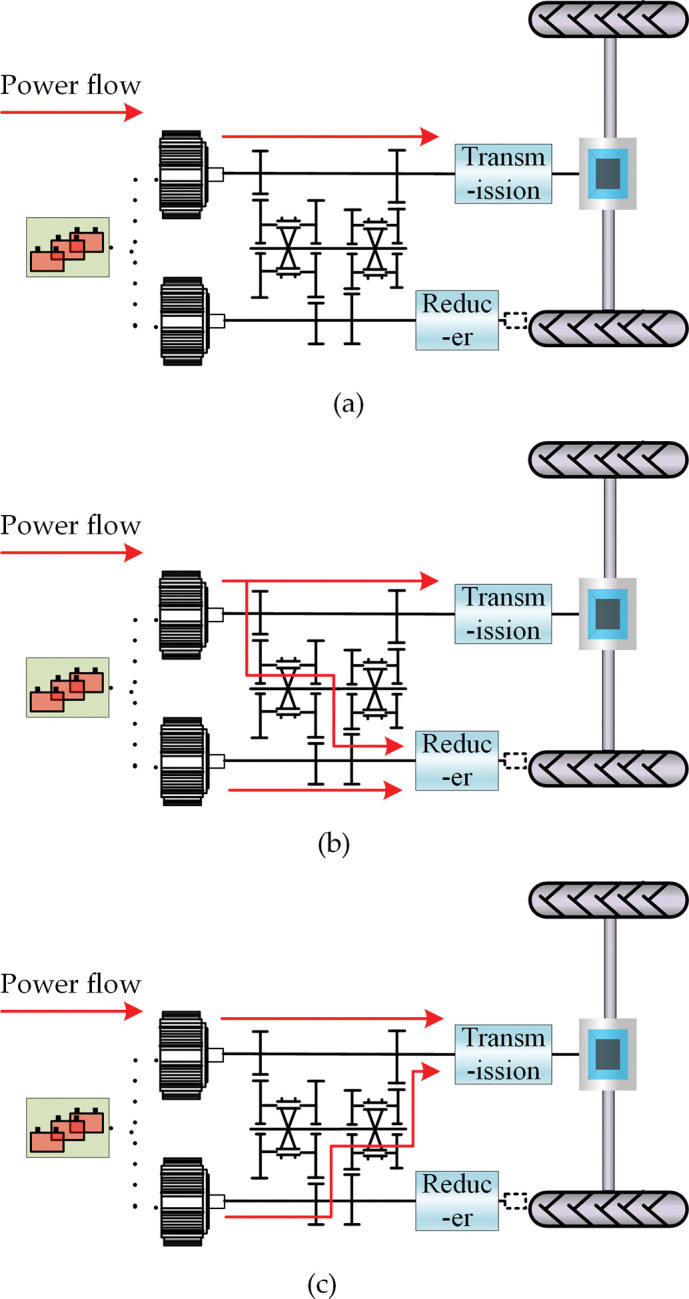
Power flow under different driving modes. (a) Power flow in single-motor drive mode. (b) Power flow in dual-motor coupling drive mode. (c) Power flow in dual-motor coupling rotary tillage mode.

When the dual-motor electric tractor drive system is in the single-motor drive mode, the traction motor is turned on, the PTO motor is turned off, and the synchronizer one and two are separated. The traction motor independently drives the dual-motor electric tractor. The power flow in this mode is shown in Fig 2(A), and its dynamic model is as follows:

$$\left\{ \begin{array}{l} T_{m}i_{g}i_{0}\eta_{d}-T_{q}=I_{m}{\dot{\omega }}_{m}i_{g}i_{0}+I_{w}{\dot{\omega }}_{w}\\ T_{q}=(F_{T}+m_{s}gf\cos\theta +m_{s}g\sin\theta )r_{w}\\ n_{m}=n_{w}i_{g}i_{0} \end{array}\right.$$ (1)

where *T*_*m*_ is the output torque of the traction motor, *i*_*g*_ is the transmission speed ratio, *i*_0_ is the central transmission device speed ratio, *η*_*d*_ is the transmission efficiency of rear drive system, *T*_*q*_ is the final drive output torque, *I*_*m*_ is the moment of inertia of the traction motor, *ω*_*m*_ is the traction motor angular velocity, *I*_*w*_ is the moment of inertia of the driving wheel, *ω*_*w*_ is the drive wheel angular velocity, *F*_*T*_ is the hook traction force, *m*_*s*_ is the maximum service mass, *g* is the gravity acceleration, *f* is the rolling friction coefficient, *θ* is the incline angle of the road, *r*_*w*_ is the drive wheel radius, *n*_*m*_ is the traction motor speed, and *n*_*w*_ is the driving wheel speed.

When the dual-motor electric tractor drive system is in the dual-motor coupling drive mode, the traction motor and PTO motor work together, synchronizer one and two engage with gears 7 and 10 respectively to the right, and the power provided by the PTO motor is coupled with the power provided by the traction motor through the transmission gear pairs 6/7 and 10/11 and outputted. The power flow in Fig 2(B) represents the coupled driving mode with two motors. In this mode, the dynamic model is as follows:

$$\left\{ \begin{array}{l} (T_{m}+T_{p}i_{pc}\eta_{pc})i_{g}i_{0}\eta_{d}-T_{q}=(I_{m}{\dot{\omega }}_{m}+I_{p}{\dot{\omega }}_{p}i_{pc})i_{g}i_{0}+I_{w}{\dot{\omega }}_{w}\\ n_{m}=\frac{n_{p}}{i_{pc}}=n_{w}i_{g}i_{0} \end{array}\right.$$ (2)

where *T*_*p*_ is the output torque of the PTO motor, *i*_*pc*_ is the power coupling device speed ratio, *η*_*pc*_ is the transmission efficiency of the power coupling device, *I*_*P*_ is the moment of inertia of the PTO motor, *ω*_*P*_ is the PTO motor angular velocity, and *n*_*p*_ is the PTO motor speed.

When the dual-motor electric tractor drive system is in the dual-motor coupling rotary tillage mode, the traction motor and PTO motor are both in the working state. Synchronizers one and two simultaneously engage with gears 5 and 8, respectively, and the power provided by the traction motor is coupled with the power provided by the PTO motor through transmission gears 4/5 and 8/9 and outputted. The power flow in this mode is shown in Fig 2(C), and its dynamic model is as follows:

$$\left\{ \begin{array}{l} T_{m1}i_{g}i_{0}\eta_{d}-T_{q}=I_{m1}{\dot{\omega }}_{m}i_{g}i_{0}+I_{w}{\dot{\omega }}_{w}\\ (T_{m2}i_{pc}\eta_{pc}+T_{p})i_{r}\eta_{r}-T_{PTO}=I_{m2}{\dot{\omega }}_{m}i_{pc}i_{r}+I_{p}{\dot{\omega }}_{p}i_{r}\\ n_{p}=\frac{n_{m}}{i_{pc}}=n_{PTO}i_{r} \end{array}\right.$$ (3)

where *T*_*m*1_ is the traction motor sent to the transmission, *I*_*m*1_ is the moment of inertia of the traction motor in the direction of *T*_*m1*_, *T*_*m*2_ is the torque delivered by the traction motor to the PTO, *I*_*m*2_ is the moment of inertia of the traction motor in the direction of *T*_*m2*_, *i*_*r*_ is the speed ratio of the reducer, *η*_*r*_ is the transmission efficiency of the reducer, *T*_*TPO*_ is the PTO output torque, and *n*_*PTO*_ is the PTO output speed.

### Motor model

The traction motor and PTO motor both use permanent magnet synchronous motors, which have good power and economic performance compared to other motors. The efficiency of the motor will affect the overall energy efficiency of the vehicle’s operation. The efficiency model of the two motors can be established using the quasi-static chart of output speed and torque, and the motor efficiency can be obtained by looking up the table. The relationship between motor efficiency and speed and torque is as follows:

η=f(n,T) (4)

where *n* is the motor speed, *T* is the motor torque, and *η* is the motor operating efficiency.

The power consumption of a motor can be expressed as follows:

$$P=\frac{nT}{9550\eta }$$ (5)

where *P* is the motor power.

### Power battery model

This paper adopts the commonly used internal resistance model in the parameter design of electric vehicles as the battery model [25].

The voltage characteristic equation of a power battery is as follows:

$$U_{b}=E_{b}-I_{b}R_{b}$$ (6)

where *U*_*b*_ is the load voltage, *E*_*b*_ is the electromotive force of the battery, *I*_*b*_ is the battery current, and *R*_*b*_ is the internal resistance of the battery.

The power expression is as follows:

$$P_{bat}=I_{b}U_{b}$$ (7)

where *P*_*bat*_ is the battery output power.

By combining Eqs (6) and (7), the total current in the circuit can be obtained as follows:

$$I_{b}=\frac{E_{b}-\sqrt{E_{b}^{2}-4R_{b}P_{bat}}}{2R_{b}}$$ (8)

In this paper, the battery SOC is calculated by the ampere-hour integral method [26].

$$SOC(t_{s})=SOC_{0}-\frac{\int_{0}^{t_{s}}I_{b}(t_{s})dt}{C_{b}}$$ (9)

where *SOC* (*t*_*s*_) is the state of charge of the battery at time *t*_*s*,_ *SOC*_*0*_ is the state of charge of the battery at the initial moment, and *C*_*b*_ is the battery capacity.

### Tire model

The *Dugoff* tire model is a steady-state model in the theoretical model, which comprehensively considers parameters such as tire stiffness and slip rate. The model has the advantages of simplicity, a small number of parameters and easy real-time application. In this paper, the *Dugoff* tire model is used to calculate the driving force [27], and the driving force of the driving wheels is as follows:

$$F_{q}=\{\begin{array}{l} F_{Z}[\varphi -\varphi^{2}\frac{F_{Z}(1-\zeta )}{4c\zeta }],\;\frac{c\zeta }{1-\zeta }\geq \frac{\varphi F_{Z}}{2}\\ \frac{c\zeta }{1-\zeta },\frac{c\zeta }{1-\zeta }\leq \frac{\varphi F_{Z}}{2} \end{array}$$ (10)

where *F*_*q*_ is the driving force of the driving wheel, *F*_*Z*_ is the load on the driving wheel, *φ* is the slip ratio of the drive wheels, *ζ* is the adhesion coefficient of the drive wheels, *c* is the horizontal distance from the hook traction point to the center of the rear wheels.

## Main parameter design

The main working conditions of the tractor are plowing and rotary tillage, and the resistance loads are quite different under different working conditions. The performance of the dual-motor electric tractor is closely related to parameters such as the power of the dual-motor, the transmission speed ratio, and the maximum service mass. Correct selection of these parameters can effectively improve the drive and economy of the dual-motor electric tractor. The basic parameters and performance indicators of the dual-motor electric tractor are shown in Table 1 [28].

**Table 1:** Main technical parameters of the reference tractor.

Project	Parameter	Value
Basic parameters	Driving wheel rolling radius	0.6 m
	Coefficient of rolling resistance	0.1
	Reducer ratio	5.56
	Central transmission device ratio	26.36
Design specifications	Rated traction	13.5 kN
	Working hours	≥1.5 h

### Motor parameter design

In order to meet the driving requirements of the tractor, the total rated power of the dual motors should meet the demand for rated traction force. In farmland operations, plowing is the most common operation with the heaviest load. Therefore, the determination of the rated traction force should firstly meet the needs of plowing operations. The average resistance of farm implements during plowing operations is as follows:

$$F_{L}=zb_{l}h_{l}k_{l}$$ (11)

where *z* is the number of plowshares, *b*_*l*_ is the single plowshare width, *h*_*l*_ is plowing deep, and *k*_*l*_ is the soil specific resistance.

Considering that the load fluctuates greatly during farmland operations, a reserve of 10% to 20% should be reserved [29], so the rated traction force of the tractor is as follows:

$$F_{TN}=(1.1∼1.2)F_{L}$$ (12)

Where *F*_*TN*_ is the rated traction.

The total rated power of the dual motors should meet the power required by the tractor for plowing operations, that is:

$$P_{TN}=\frac{F_{TN}v_{T}}{3600\eta_{T}}$$ (13)

Where *P*_*TN*_ is the sum of the rated power of the two motors, *v*_*T*_ is the tractor plowing operation speed, and *η*_*T*_ is the transmission efficiency of the whole machine.

When the tractor performs rotary tillage operations, the PTO motor provides the power output of the tractor drive output shaft, and the power required for rotary tillage operations is as follows [30]:

$$P_{P}=k_{p}h_{p}v_{p}B/36$$ (14)

where *P*_*P*_ is the average power consumption of rotary tillage, *k*_*p*_ is the specific resistance of rotary tillage, *h*_*p*_ is the depth of rotary tillage, *v*_*p*_ is the tractor rotary tillage speed, and *B* is working width.

Considering that the load fluctuates during the rotary tillage operation, the rated power of the PTO motor should meet the following conditions [24].

$$P_{PN}=1.2\frac{P_{p}}{\eta_{T}}$$ (15)

where *P*_*PN*_ is the rated power of the PTO motor.

The rated power of the PTO motor is calculated by Eq (14) so the rated power of the traction motor is as follows:

$$P_{MN}=P_{TN}-P_{PN}$$ (16)

Where *P*_*MN*_ is the rated power of the traction motor.

**Fig 3:**
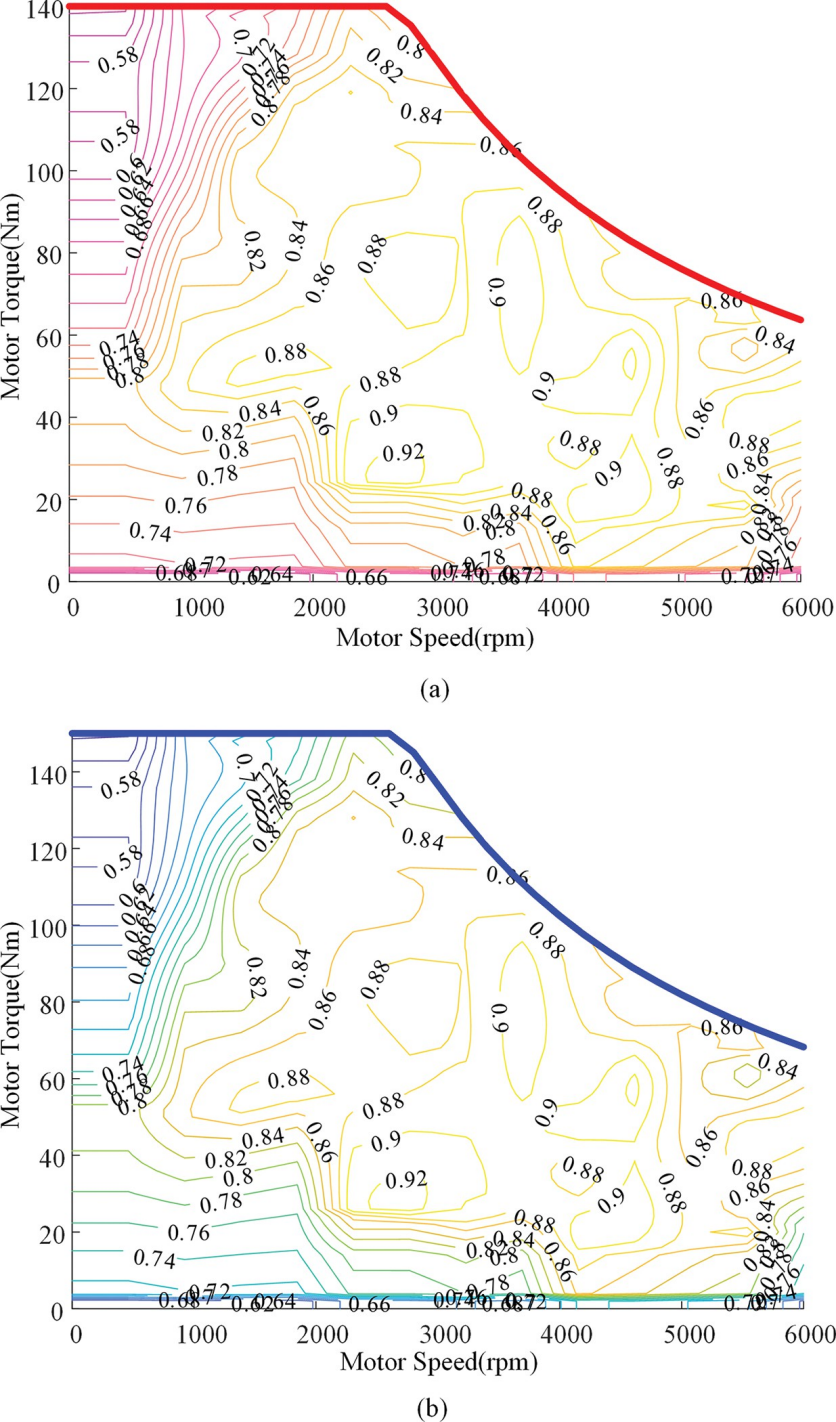
Efficiency MAP of motor. (a) Traction motor. (b) PTO motor.

According to the above analysis and calculation, the specific parameters of traction motor and PTO motor are shown in Table 2. The MAP graph of the two motors is shown in Fig 3.

**Table 2:** The main parameters of the motor.

Motor	parameter	Value
Traction motor	Rated/peak power	19/42 kW
	Rated/maximum torque	65/140 N·m
	Rated/maximum speed	2800/6000 r·min^-1^
PTO motor	Rated/peak power	20/45 kW
	Rated/maximum torque	70/150 N·m
	Rated/maximum speed	2800/6000 r·min^-1^

### Transmission gear setting

Dual-motor electric tractors are widely used and have different requirements for driving speed and driving torque. When the tractor is in the working state, it works with the constant power characteristic of the motor at high speed, so that it has a large operating speed range. Therefore, the calculation of the speed ratio can set the transmission gear according to the load under different working conditions. According to the different load rates of tractors, the load conditions of tractors are divided into two types: 100% load rate and 55% load rate [31]. One kind of work load corresponds to one gear ratio.

The plowing and other heavy-duty operations of the electric tractor are defined as 100% load rate. At this time, the transmission is in the low gear. In order to ensure that the drive system can provide sufficient power, the speed ratio of the drive system is as follows:

$$\left\{ \begin{array}{l} i_{d}{}_{1}=\frac{F_{TN}r_{w}}{T_{\max}\omega_{m}\eta_{T}}\\ \omega_{m}=\frac{v_{T}i_{d1}}{r_{w}}\\ i_{g1}=\frac{i_{d1}}{i_{0}} \end{array}\right.$$ (17)

Where *i*_*d*1_ is the total speed ratio of the low gear of the rear drive system, *T*_*max*_ is the maximum torque output by the power coupling device, and *i*_*g*1_ is the transmission low ratio.

Therefore, the transmission high gear ratio can be determined, that is:

$$i_{g2}=0.55i_{g1}$$ (18)

where *i*_*g*2_ is the transmission high ratio.

After the above analysis and calculation, the speed ratio of the low-speed gear of the dual-motor electric tractor is 3.32, and the speed ratio of the high-speed gear is 1.62.

### Mass parameter design

The maximum service mass of a tractor is closely related to the driving performance and energy consumption index of the whole machine. At the time of design, the maximum service mass depends on the rated traction force and soil conditions under the operating conditions, that is, the rated traction force can be exerted under the condition that the slip rate does not exceed the specified value [31].

$$m_{s}=\frac{F_{TN}}{\varphi_{\sigma }\lambda -f}$$ (19)

where *φ*_*σ*_ is the coefficient of adhesion when the slip rate is a specified value, *λ* is the dynamic mass partition coefficient.

Based on the above analysis and calculation, the maximum service mass of the dual-motor electric tractor is 2210kg.

## Parameter optimization method based on driving cycles

The power coupling device and power battery are the core components of the dual-motor electric tractor. The selection of its parameters not only affects the driving performance of the tractor during field operations, but also affects the economic performance of the whole machine. After the design of the main parameters, the rated power of the dual motors, the transmission speed ratio and the maximum service mass can be obtained, but the parameters of the power coupling device and the power battery have not been determined. This paper proposes the POMBDC, that is, the torque distribution of the traction motor and the PTO motor is carried out by using a control strategy based on instantaneous optimization. On this basis, the power coupling device speed ratio and the power battery capacity are optimized. This method can realize simultaneous optimization of the power coupling device speed ratio and the power battery capacity, so as to improve the operating efficiency of the whole machine and reduce energy consumption.

### Control strategy based on instantaneous optimization

When the dual-motor electric tractor works in the single-motor drive mode, the operating point of the traction motor is directly determined by the required operating speed and required torque, so this mode does not need to be optimized. When the dual-motor electric tractor is in the dual-motor coupling drive and dual-motor coupling rotary tillage modes, the torque of the two motors has multiple distribution methods under constraint conditions, and the power consumption of the motors at different operating points is different. Reasonably optimize the dual-motor output torque, which can effectively improve the operating efficiency of the motor.

From Eqs (2) and (3), it can be seen that the resistance of the dual-motor electric tractor is different in different modes. When the dual-motor tractor is in the dual-motor coupling driving mode, the required torque and required speed at the output end of the power coupling device are as follows:

$$\left\{ \begin{array}{l} T_{v}=\frac{T_{q}+I_{w}{\dot{\omega }}_{w}+(I_{m}{\dot{\omega }}_{m}+I_{p}{\dot{\omega }}_{p}i_{pc})i_{g}i_{0}}{i_{g}i_{0}\eta_{T}}\\ N_{v}=\frac{i_{g}i_{0}v_{T}}{0.377r_{w}} \end{array}\right.$$ (20)

where *T*_*v*_ is the required torque at the output end of the power coupling device, *N*_*v*_ is the required speed at the output end of the power coupling device.

When the dual-motor tractor is in the dual-motor coupling rotary tillage mode, the required torque and required speed at the output end of the power coupling device are as follows:

$$\left\{ \begin{array}{l} T_{v}=\frac{T_{q}+I_{w}{\dot{\omega }}_{w}+I_{m1}{\dot{\omega }}_{m}i_{g}i_{0}}{i_{g}i_{0}\eta_{d}}+\frac{T_{PTO}+I_{p}{\dot{\omega }}_{p}i_{r}+I_{m2}{\dot{\omega }}_{m}i_{pc}i_{r}}{i_{r}\eta_{r}}\\ N_{v}=n_{PTO}i_{r} \end{array}\right.$$ (21)

In the optimization process, the sum of the power consumption of the two motors is used as the objective function, that is:

$$\begin{array}{l} L=P_{m}+P_{p}\\ \;=\frac{T_{m}n_{m}}{9550\eta_{m}}+\frac{T_{p}n_{p}}{9550\eta_{p}} \end{array}$$ (22)

where *L* is the sum of the power consumption of the two motors, *P*_*m*_ is the traction motor power, *P*_*p*_ is the PTO motor power, *η*_*m*_ is the traction motor efficiency, and *η*_*p*_ is the PTO motor efficiency.

According to the characteristics of the motor components, the constraints are as follows:

$$\left\{ \begin{array}{l} T_{m\min}\leq T_{m}\leq T_{m\max}\\ N_{m\min}\leq N_{m}\leq N_{m\max}\\ T_{p\min}\leq T_{p}\leq T_{p\max}\\ N_{p\min}\leq N_{p}\leq N_{p\max} \end{array}\right.$$ (23)

where *T*_*mmin*_ and *T*_*mmax*_ are the minimum and maximum torque of the traction motor, respectively; *N*_*mmin*_ and *N*_*mmax*_ are the minimum and maximum speeds of the traction motor, respectively*; T*_*pmin*_ and *T*_*pmax*_ are the minimum and maximum torque of the PTO motor, respectively; *N*_*pmin*_ and *N*_*pmax*_ are the minimum and maximum speeds of the PTO motor, respectively.

**Fig 4:**
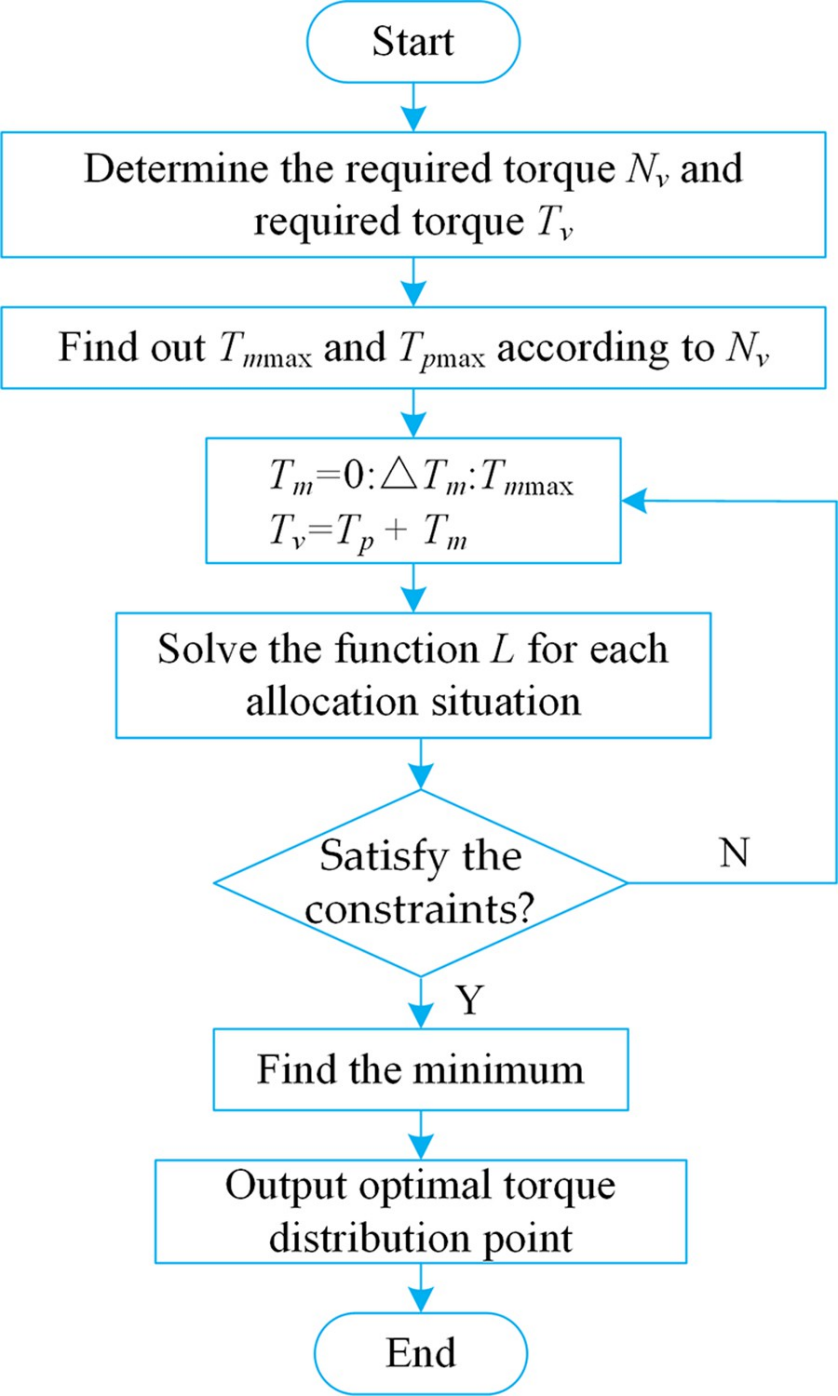
The flow chart of instantaneous optimization solution.

The solution process is shown in Fig 4.

The detailed solution process of the instantaneous optimization shown in Fig 4 is as follows. First, determine the required torque and required speed at the output end of the power coupling device. Next, find out the maximum torque of the traction motor and PTO motor according to the required speed, and then distribute the torque to the traction motor and PTO motor. After solving the value of the function *L* for each allocation situation, judge whether the exit condition is satisfied, if so, solve the minimum value *L* of the sum of the power of the two motors, if not, then perform torque distribution again, until all torque distribution methods are traversed. The final output torque distribution when the sum *L* of the power of the dual motors is the minimum value.

### Drive system parameter optimization

The power coupling device speed ratio and the power battery capacity have a great influence on the economy of the whole machine. This section optimizes the above parameters. In order to verify the influence of the speed ratio *i*_*pc*_ of the power coupling device on the economy of the whole machine, this paper sets the value range of *i*_*pc*_ as (1, 4.0) [32].

The total power consumption of the dual-motor electric tractor is as follows:

$$Q_{c}=f(P_{m},P_{p},\eta_{ba},i_{pc})$$ (24)

where *Q*_*c*_ is the total power consumption of the dual-motor electric tractor, *η*_*ba*_ is the power battery discharge efficiency.

Under different speed ratios of power coupling devices, the power consumption for completing specific working conditions is different. In order to ensure the power and economy of the whole machine, the lowest power consumption is selected as the capacity of the power battery. The power battery capacity is as follows.

$$Q_{b}=\min(Q_{c})$$ (25)

where *Q*_*b*_ is the power battery capacity.

**Fig 5:**
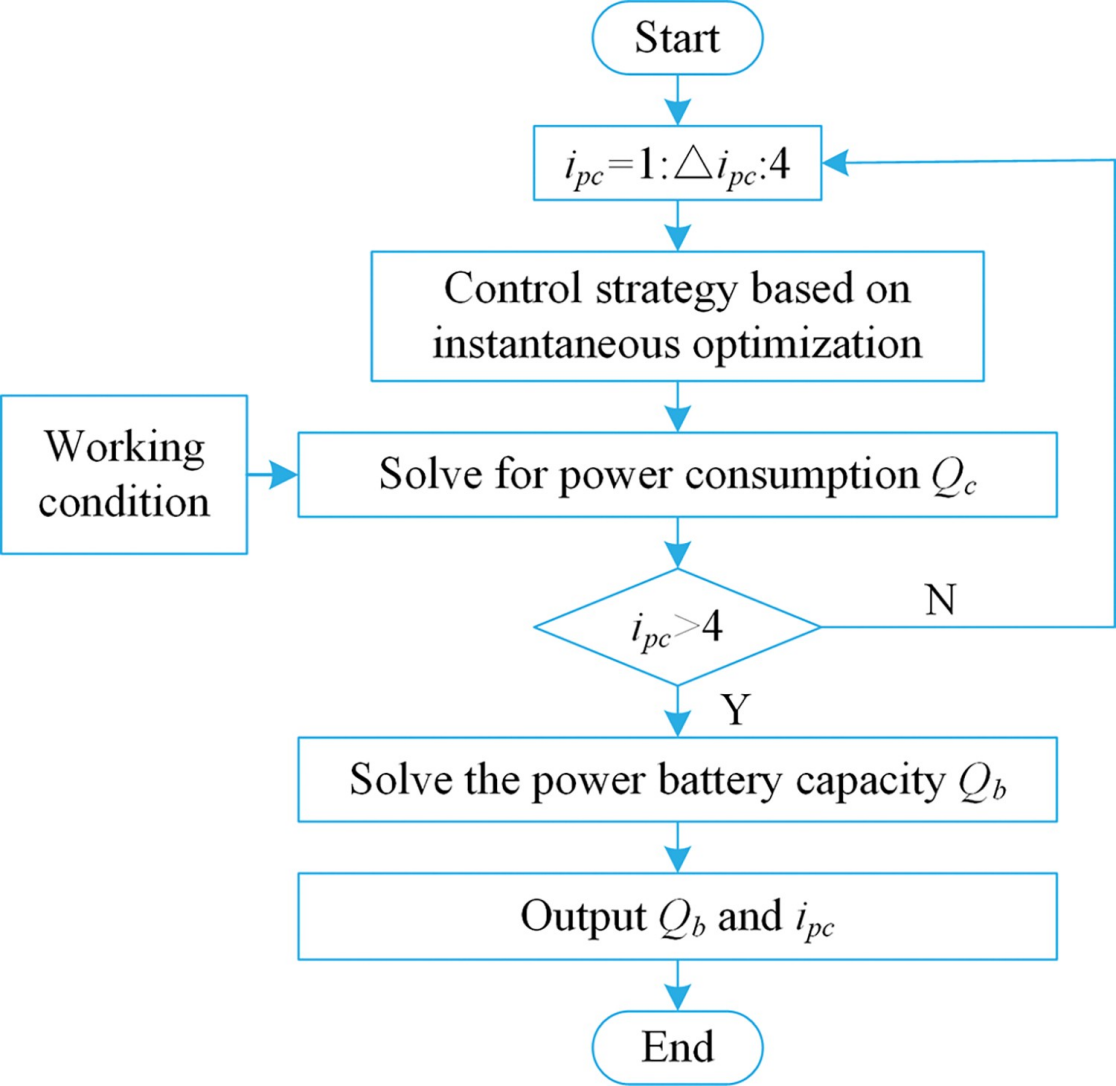
The flow chart of drive system parameter optimization.

The optimization process is shown in Fig 5.

The detailed solution process of the drive system parameter optimization shown in Fig 5 is as follows. First, the power coupling device speed ratio is assigned, and the control strategy based on instantaneous optimization is used to optimize the torque of the dual motors under the current speed ratio. Next, input the actual working condition information to solve the power consumption *Q*_*c*_. After traversing the power coupling device speed ratio, the minimum value of power consumption *Q*_*c*_ is calculated as the power battery capacity *Q*_*b*_. Finally, output the power coupling device speed ratio and the power battery capacity.

## Comparative method design and simulation analysis

### Comparative method design

The POMBDC refers to the optimization of the dual-motor torque distribution method using a control strategy based on instantaneous optimization. On this basis, the power coupling device speed ratio and the power battery capacity are optimized. To verify the advantages of the proposed POMBDC, this paper formulates the instantaneous optimization-constant speed ratio design method (IO-CSRDM), the rule-optimized speed ratio design method(R-OSRDM) and the rule-constant speed ratio design method(R-CSRDM) as comparison methods. The IO-CSRDM means that the control strategy based on instantaneous optimization is used to control the motor, but only the power battery capacity is optimized, and the power coupling device speed ratio is no longer optimized. In the design process, the power coupling device speed ratio is set to 1. The R-OSRDM is to the use the power following control strategy to control the dual motors, and optimize the power coupling device speed ratio and the power battery capacity. The R-CSRDM refers to adopting the power following control strategy and setting the power coupling device speed ratio as 1, only optimizing the power battery capacity.

### Simulation analysis

In this section, based on the Matlab simulation platform, the four methods are simulated and analyzed under plowing conditions and rotary tillage conditions.

**Fig 6:**
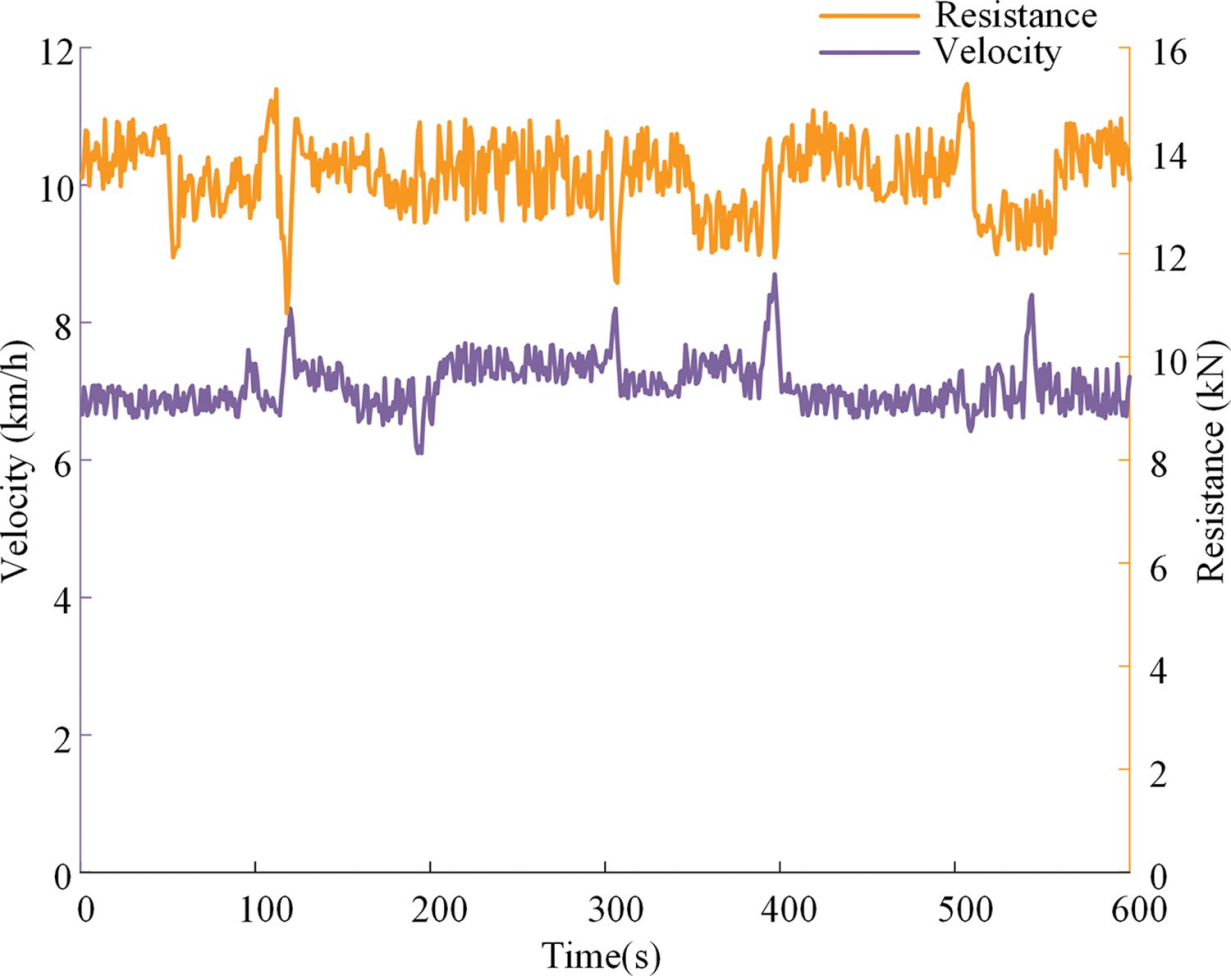
Variation curves of operating speed and traction resistance under plowing conditions.

**Simulation experiment of plowing conditions.** During the plowing operation of the tractor, the resistance will change with the plowing depth and soil specific resistance. Fig 6 shows the actual operation speed and plowing resistance collected during the tractor plowing operation. Among them, the width of the share plough pulled by the tractor is 250mm, the number of ploughs per operation is 3, the average tillage depth is 216 mm, the average operation speed is 7.08km/h, and the operating time is 600s.

When the electric tractor is in the dual-motor coupling drive mode, after inputting the plowing condition information into the POMBDC, the optimal speed ratio of the power coupling device is 1.63. The power coupling device speed ratio obtained by using the R-OSRDM is 1.34.

**Fig 7:**
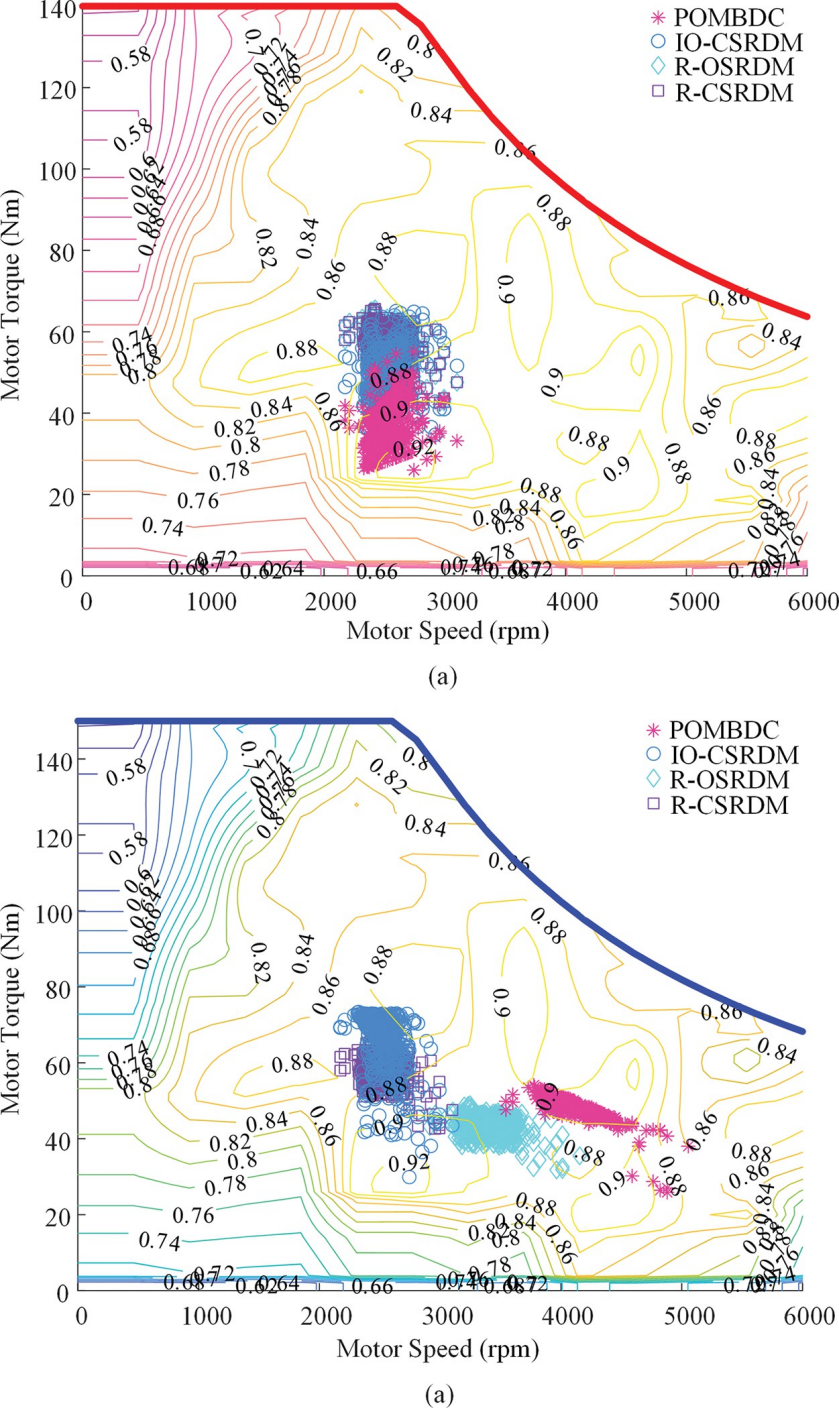
Two motor operating points of four methods under plowing conditions. (a)Working point of traction motor. (b) Working point of PTO motor.

Fig 7 shows the distribution of the working points of the two motors in the four methods under plowing conditions. It can be seen from the figure that, compared with the comparison method, the POMBDC has fewer operating points in the low-efficiency area of the dual motors, which is conducive to improving the efficiency of the whole machine and reducing energy consumption.

**Fig 8:**
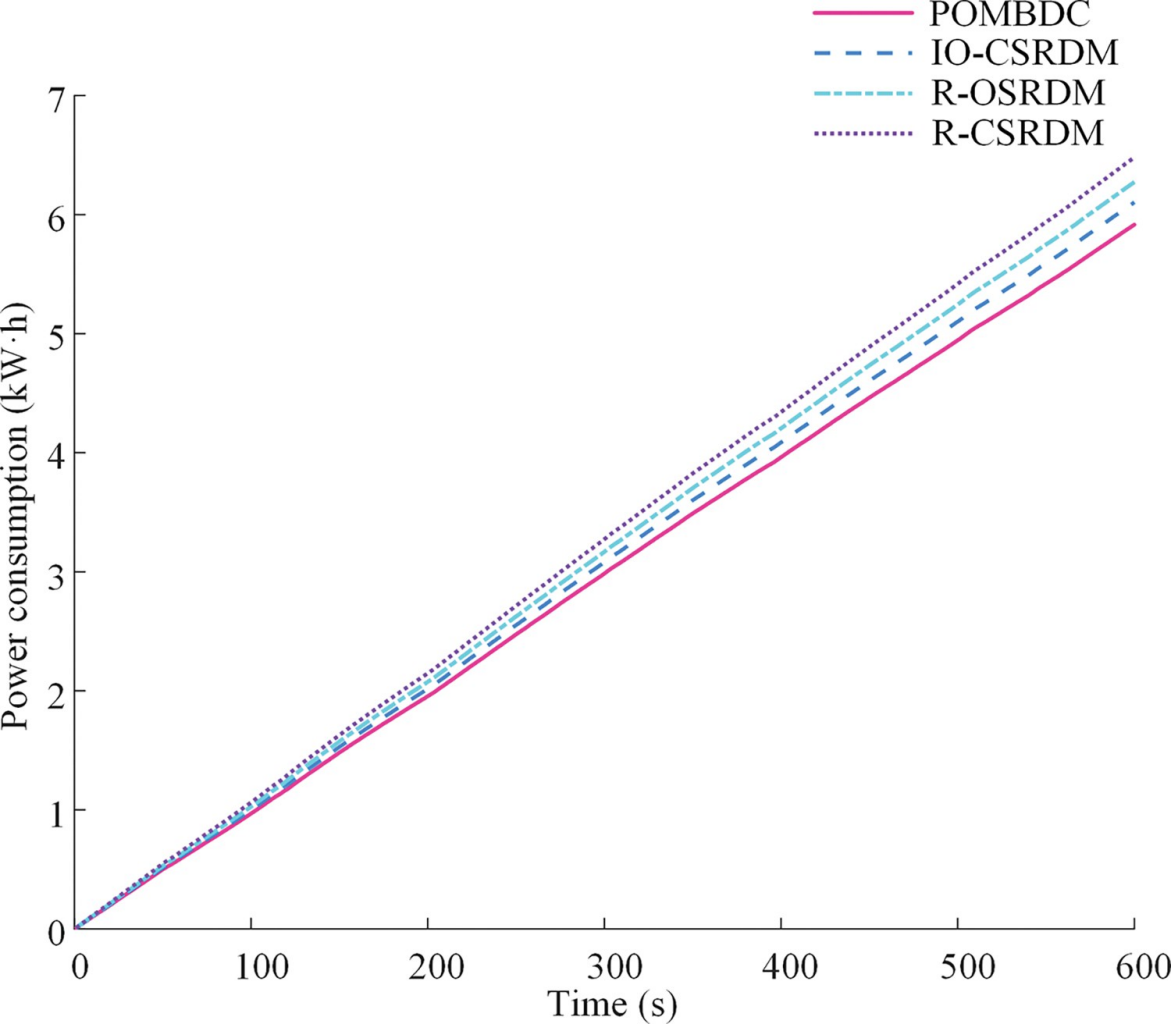
Power consumption of four methods under plowing conditions.

Fig 8 shows the change of power consumption over time for the four methods under plowing conditions. It can be seen from the figure that the POMBDC is the smallest among the four methods. The power consumption of the POMBDC, IO-CSRDM, R-OSRDM, and R-CSRDM are 5.91 kW·h, 6.10 kW·h, 6.27 kW·h, and 6.48 kW·h, respectively. The power consumption resulting from the POMBDC is reduced by 3.11%, 5.74%, and 8.8%, compared with those of the IO-CSRDM, R-OSRDM, and R-CSRDM, respectively.

**Fig 9:**
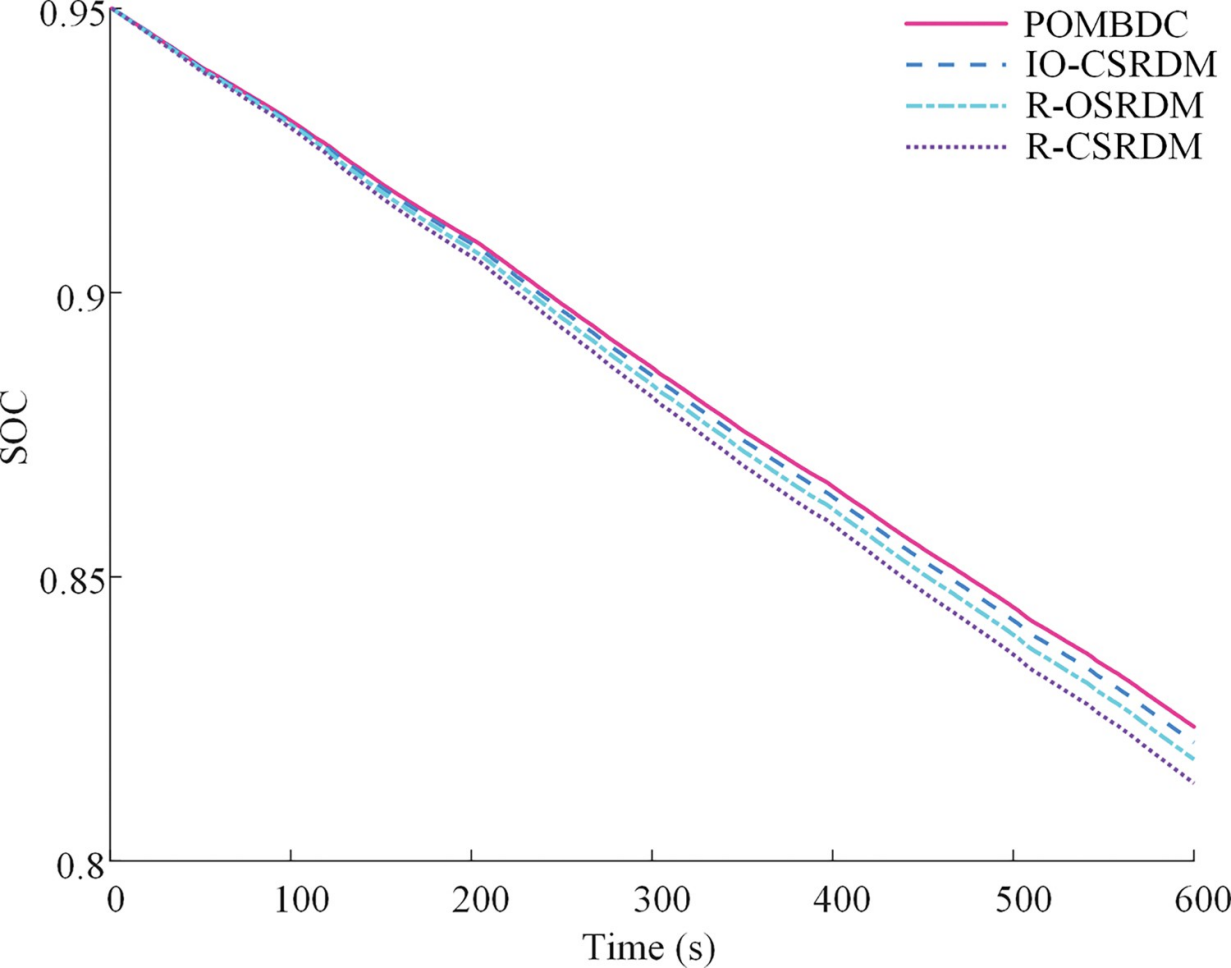
Power battery SOC of four methods under plowing conditions.

Fig 9 shows the time history of the power battery SOC resulting from the four methods under plowing conditions. The initial SOC value is 0.95. The power battery SOCs of the POMBDC, IO-CSRDM, R-OSRDM, and R-CSRDM decrease by 12.64%, 12.91%, 13.21%, and13.63%, respectively. The minimum change in SOC can be obtained under the POMBDC.

Table 3 shows the parameters of power battery and power coupling device obtained by four methods under plowing conditions. Under the premise of satisfying the design index, the power battery capacity of the dual-motor electric tractor obtained by using the POMBDC is 59.16kW·h, which is 3.08% lower than using the IO-CSRDM, 5.71% lower than using the R-OSRDM, and 8.73% lower than using the R-CSRDM. Using the POMBDC can effectively avoid the waste of resources and increase the cost caused by the excessive capacity of the power battery.

**Table 3:** Parameters of power battery and power coupling device obtained by different methods.

Name	Parameter	Value
POMBDC	Power battery capacity	59.16 kW·h
	Power coupling device speed ratio	1.63
IO-CSRDM	Power battery capacity	61.04 kW·h
	Power coupling device speed ratio	1.00
R-OSRDM	Power battery capacity	62.74 kW·h
	Power coupling device speed ratio	1.34
R-CSRDM	Power battery capacity	64.82 kW·h
	Power coupling device speed ratio	1.00

**Fig 10:**
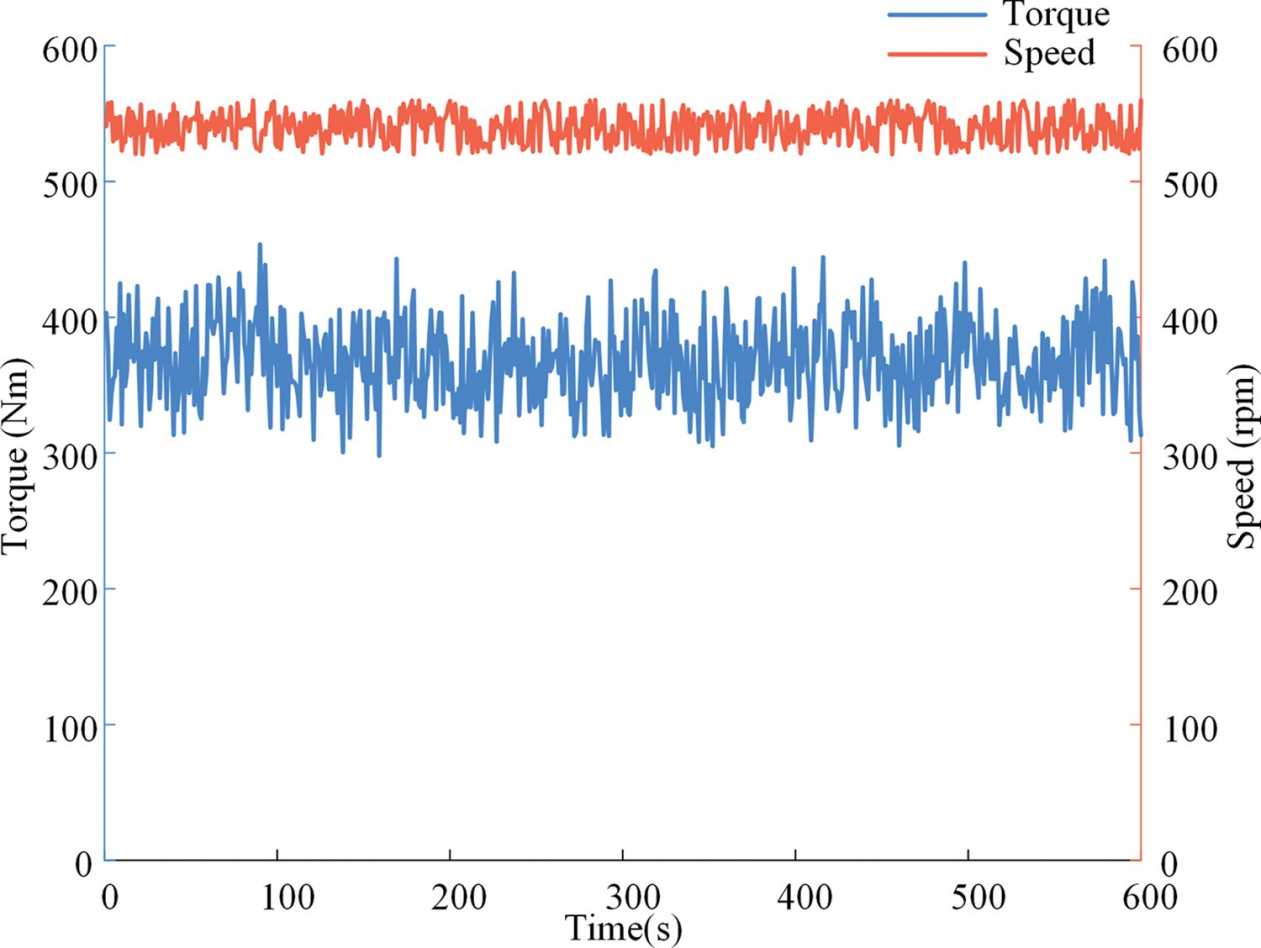
Torque and tachograph of the PTO.

Simulation experiment of rotary tillage operation conditions. In addition to plowing conditions, rotary tillage is another major working condition of tractors. When the tractor is in the dual-motor power coupling rotary tillage mode, PTO is not affected by the tractor’s driving conditions. According to the field rotary tillage test, the torque and speed changes are shown in Fig 10. Among them, the width of the rotary tiller is 1600mm, the average tillage depth is 158 mm, and the operating time is 600s.

When the electric tractor is in the dual-motor power coupling rotary tillage mode, after inputting the condition information into the POMBDC, the optimal speed ratio of the power coupling device is 1.55. The power coupling device speed ratio obtained by using the R-OSRDM is 1.26.

**Fig 11:**
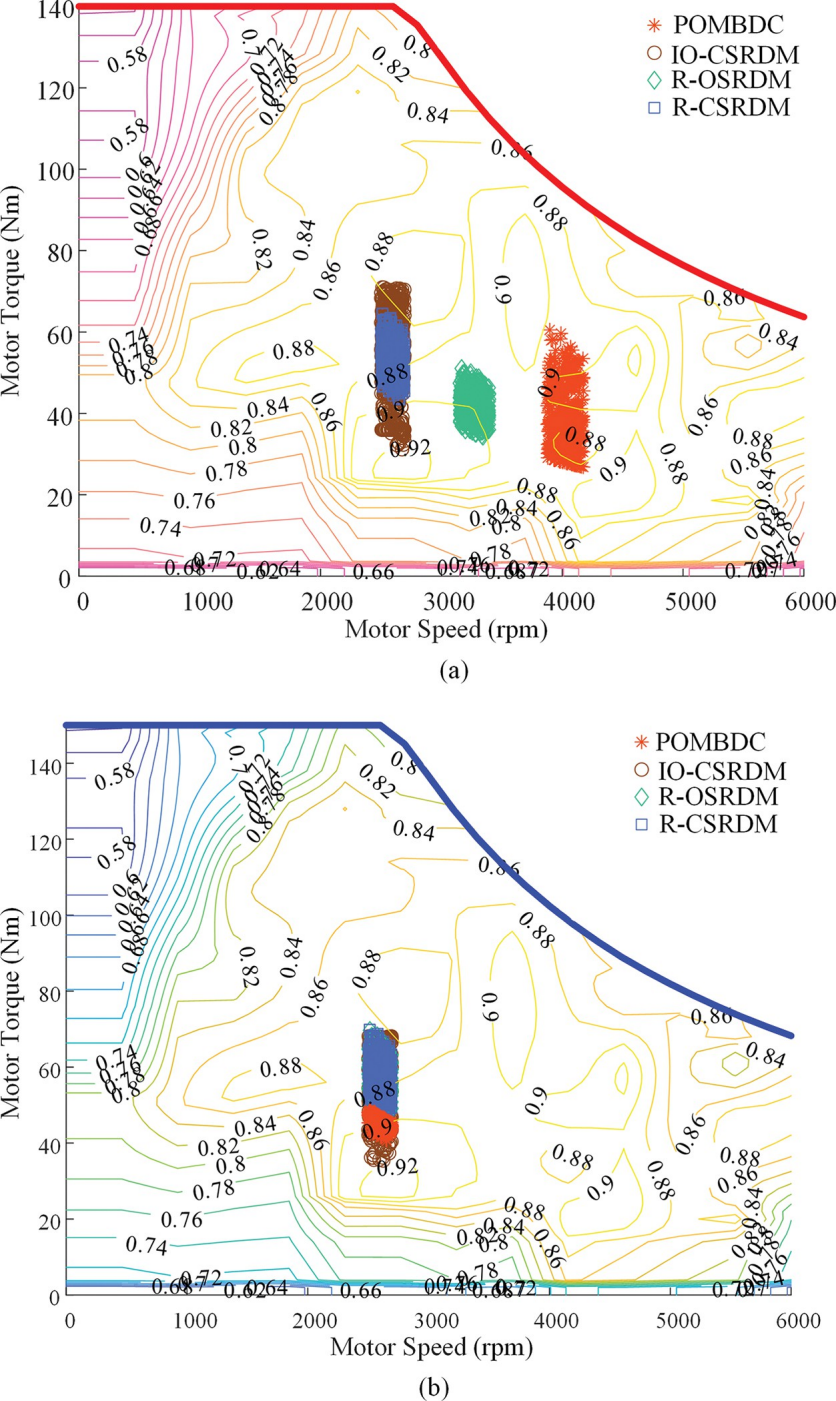
Two motor operating points of four methods under rotary tillage conditions. (a)Working point of traction motor. (b) Working point of PTO motor.

Fig 11 shows the working points of traction motors and PTO motors in the four methods under rotary tillage conditions. It can be seen from Fig 11 that after optimization by the POMBDC, the proportion of the operating points of the two motors in the high-efficiency range is larger, the distribution of the operating points of the motors is better. The efficiency of the drive system is significantly improved.

**Fig 12:**
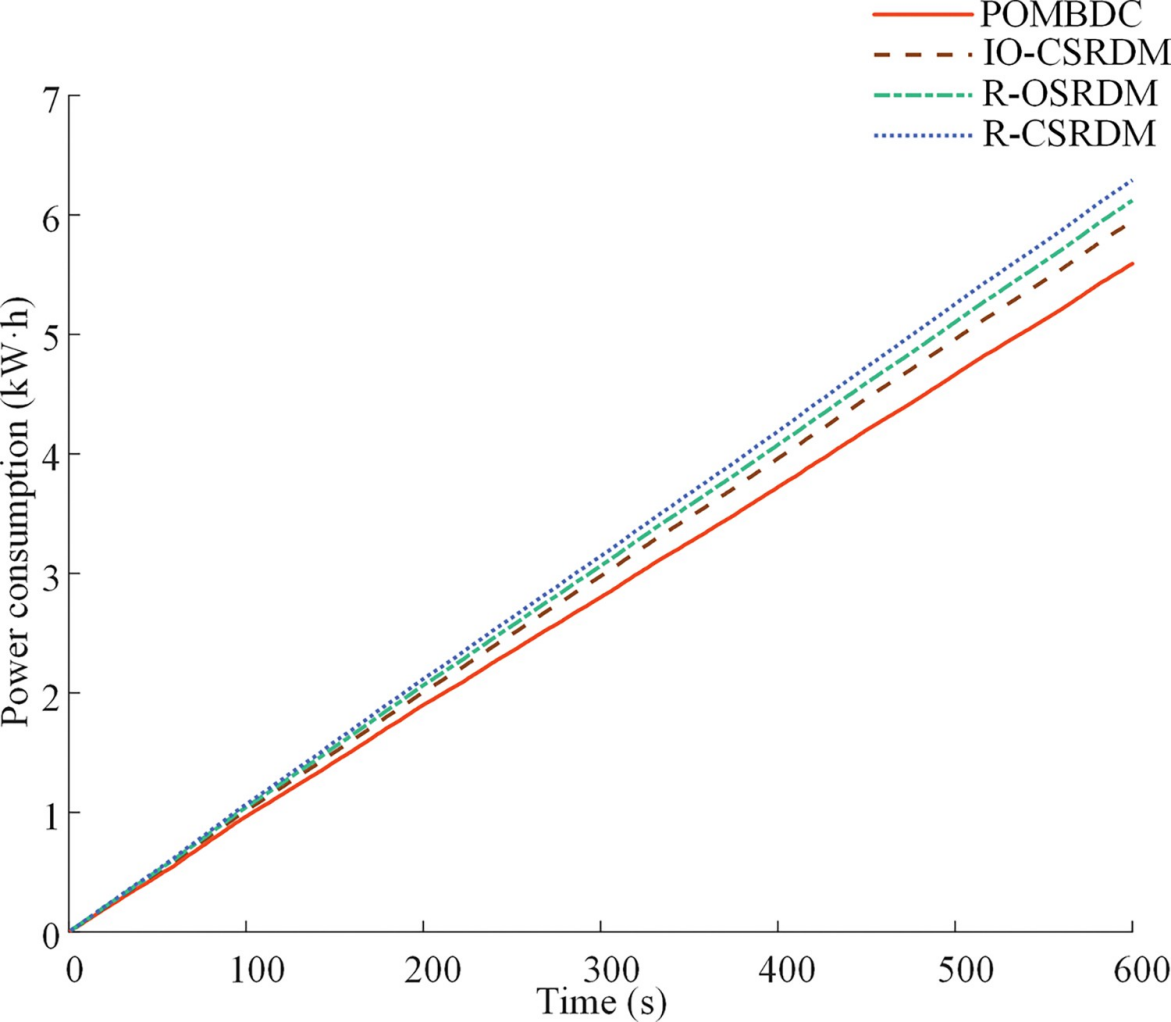
Power consumption of four methods under rotary tillage conditions.

Fig 12 shows the change of power consumption over time obtained by four methods of dual-motor electric tractor under rotary tillage conditions. We see that the power consumption of the POMBDC is the smallest among the four, with the values for POMBDC, IO-CSRDM, R-OSRDM, and R-CSRDM being 5.59 kW·h, 5.95 kW·h, 6.12 kW·h, and 6.29 kW·h, respectively. The power consumption of the POMBDC is 6.05%, 8.66%, and 11.13% lower than those of the IO-CSRDM, R-OSRDM, and R-CSRDM, respectively.

**Fig 13:**
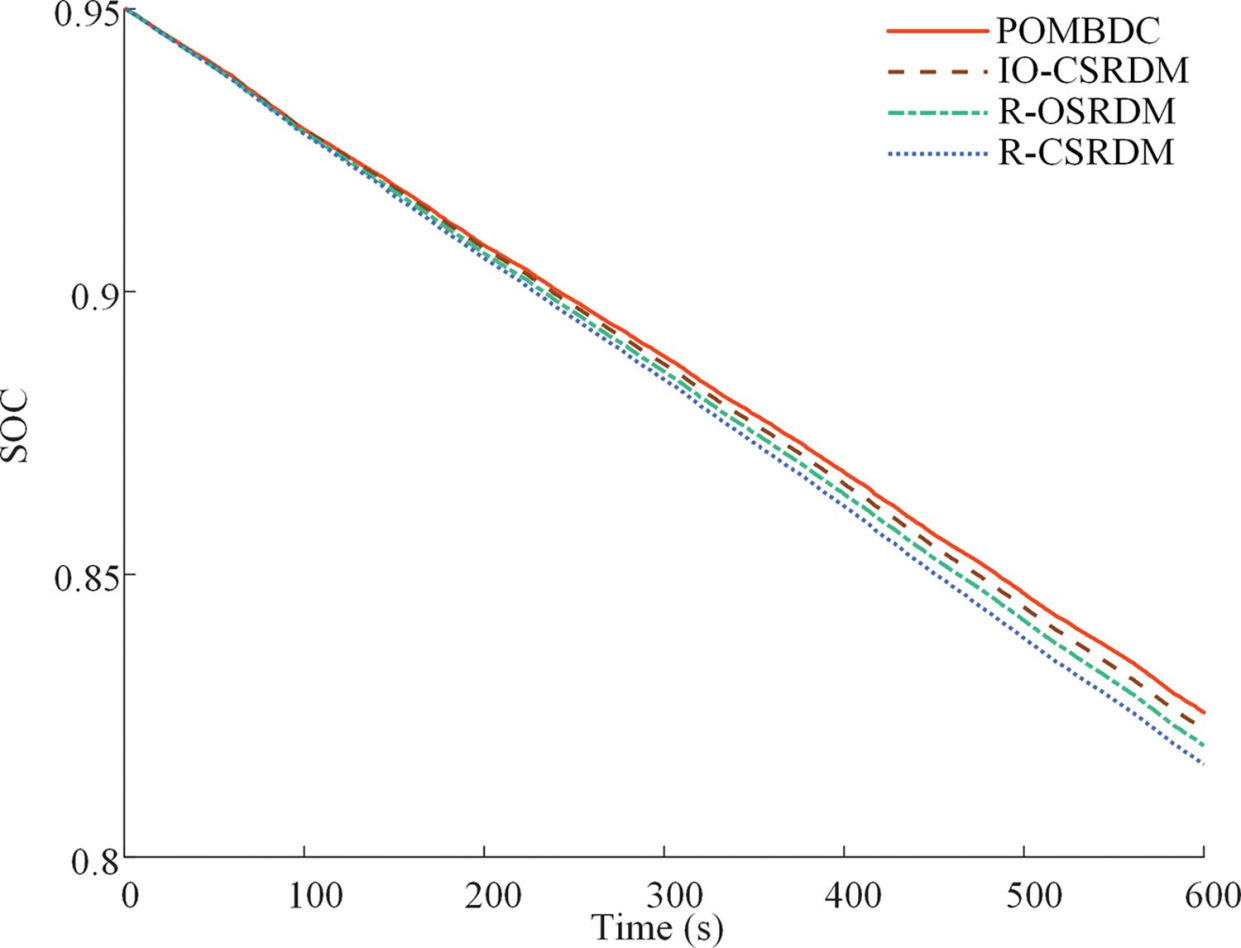
Power battery *SOC* of four methods under rotary tillage conditions.

Fig 13 shows the time history of the power battery SOC resulting from the four methods under rotary tillage conditions. The initial SOC value is 0.95. The power battery SOCs resulting from the POMBDC, IO-CSRDM, R-OSRDM, and R-CSRDM are decreased by 12.44%, 12.74%, 13.03%, and13.36%, respectively.

Table 4 shows the parameters of power battery and power coupling device obtained by using four methods under rotary tillage conditions. The power battery capacity resulting from the POMBDC is 6% lower than that obtained from the IO-CSRDM, 8.64% lower than that obtained from the R-OSRDM, and 11.11% lower than that obtained from the R-CSRDM. Under the same design index, the power battery capacity obtained by the POMBDC is the lowest, because the energy consumption economy and motor operation efficiency optimized by the POMBDC are the best among the four methods.

**Table 4:** Parameters of power battery and power coupling device obtained by different methods.

Name	Parameter	Value
POMBDC	Power battery capacity	55.92 kW·h
	Power coupling device speed ratio	1.55
IO-CSRDM	Power battery capacity	59.49 kW·h
	Power coupling device speed ratio	1.00
R-OSRDM	Power battery capacity	61.21 kW·h
	Power coupling device speed ratio	1.26
R-CSRDM	Power battery capacity	62.91 kW·h
	Power coupling device speed ratio	1.00

## Conclusion

A dual-motor drive system that can realize three driving modes is designed, the working principles in different modes are analyzed, and the dynamic models are established. According to the driving index, the design of the main parameters is completed. A control strategy based on instantaneous optimization is used to optimize the torque of the dual motors. On this basis, the POMBDC is formed, and the collaborative optimization of the power coupling device speed ratio and the power battery capacity is realized. To verify the advantages of the proposed POMBDC, we formulate three comparative methods and conduct simulation verification under plowing and rotary tillage conditions. The results are as follows:

1. Under plowing conditions, the power battery capacity of the POMBDC is 3.08%, 5.71%, and 8.73% lower than those of the IO-CSRDM, R-OSRDM, and R-CSRDM, respectively. The power consumption resulting from the POMBDC is reduced by 3.11%, 5.74%, and 8.8%, compared with those of the IO-CSRDM, R-OSRDM and R-CSRDM, respectively. The optimal power coupling device speed ratio obtained by the POMBDC is 1.63.
2. Under rotary tillage conditions, the power battery capacity of the POMBDC is 6%, 8.64%, and 11.11% lower than those of the IO-CSRDM, R-OSRDM, and R-CSRDM, respectively. The power consumption resulting from the POMBDC is reduced by 6.05%, 8.66%, and 11.13%, compared with those of the IO-CSRDM, R-OSRDM and R-CSRDM, respectively. The optimal power coupling device speed ratio obtained by the POMBDC is 1.55.

Therefore, under the same design index, the POMBDC can effectively extend the operating mileage and reduce the cost of the dual-motor electric tractor. The dual-motor electric tractor has instantaneous response characteristics during the mode switching process, which will affect the energy consumption of the whole machine. The mode switching process control can be deeply studied in the future.

## Supporting information

S1 File Torque distribution of two motors. (PDF)

## References

[1] Mao Y, Wu Y, Yan X, et al. Simulation and experimental research of electric tractor drive system based on Modelica. Plos one. 2022;17(11): e0276231. doi: 10.1371/journal.pone.0276231 36395258 PMC9671462

[2] Caban J, Vrabel J, Šarkan B, et al. Analysis of the market of electric tractors in agricultural production. MATEC Web of Conferences; 2018: EDP Sciences.

[3] Moinfar A, Shahgholi G, Gilandeh YA, et al. The effect of the tractor driving system on its performance and fuel consumption. Energy. 2020;202:117803.

[4] Zhang Y, Wu Z. Research on the spatial association network structure for innovation efficiency of China’s new energy vehicle industry and its influencing factors. Plos one. 2021;16(8):e0255516. doi: 10.1371/journal.pone.0255516 34437588 PMC8389435

[5] Yanpeng Z, Feng W, Yongfa Y, et al. Power system optimization of electric tractor based on working conditions and retired lithium ion battery. Journal of Chinese Agricultural Mechanization. 2022;43(2):104.

[6] Ghobadpour A, Mousazadeh H, Kelouwani S, et al. An intelligent energy management strategy for an off‐road plug‐in hybrid electric tractor based on farm operation recognition. IET Electrical Systems in Transportation. 2021;11(4):333–47.

[7] Chen Y, Xie B, Mao E. Electric tractor motor drive control based on FPGA. IFAC-PapersOnLine. 2016;49(16):271–6.

[8] Shen W, Zhou J, Ji C, et al. Research review about electric tractor in China. Journal of Chinese Agricultural Mechanization. 2017(10):102–7.

[9] Zhang S-l Wen C-k, Ren W, et al. A joint control method considering travel speed and slip for reducing energy consumption of rear wheel independent drive electric tractor in ploughing. Energy. 2023;263:126008.

[10] Xie B, Wu Z, Mao E. Development and prospect of key technologies on agricultural tractor. Transactions of the Chinese Society for Agricultural Machinery. 2018;49(8):1–17.

[11] Zhu Z, Lai L, Wang D, et al. Energy saving characteristics of the mechanical hydraulic tractor power system with oil electric hybrid power. Transactions of the Chinese Society of Agricultural Engineering. 2022;38(17).

[12] Kalociński T. Modern trends in development of alternative powertrain systems for non-road machinery. Combustion Engines. 2022;61(1).

[13] Daszkiewicz P, Andrzejewski M, Medwid M, et al. Analysis of the selection of chosen technical parameters of the powertrain system for a diesel-electric rail-road tractor. Combustion Engines. 2021;60(3).

[14] Li J, Wu X, Zhang X, et al. Design of distributed hybrid electric tractor based on axiomatic design and Extenics. Advanced Engineering Informatics. 2022;54:101765.

[15] Baek S-Y, Baek S-M, Jeon H-H, Kim W-S, Kim Y-S, Sim T-Y, et al. Traction performance evaluation of the electric all-wheel-drive tractor. Sensors. 2022;22(3):785. doi: 10.3390/s22030785 35161531 PMC8838039

[16] Zhang C, Zhu S, Wang J, et al. Matching design and performance analysis for driving system of solar garden tractor. Transactions of the Chinese Society of Agricultural Engineering. 2015;31(11):24–30.

[17] Fang S, Wang N, Yi K, et al. Design and performance analysis of power system for pure electric tractor. Journal of Chinese Agricultura-l Mechanization. 2017;38(1):80–4.

[18] Zhao J, Xu L, L, et al. Design for Drive System of Extended-range Electric Tractor. Journal of Agricultural Mechanization Research. 2018(11):236–40.

[19] Chen L, Han Q, Wang W, et al. Design and experiment of electric drive system for pure electric tractor. Transactions of the Chinese Society for Agricultural Machinery. 2018;49(8):388–94.

[20] Fu S, Li Z, Du Y, et al. Matching optimization for tractor powertrain based on improved NSGA一Ⅱalgorithm. Transactions of the Chinese Society for Agricultural Machinery. 2018;49(11):349–57.

[21] Wu H, Xia C. Parameter design and optimization of electric tractor transmission system based on Isight. Journal of Chongqing University of Technology (Natural Science). 2019;33(9):53–8.

[22] Chen Y, Xie B, Du Y, et al. Powertrain parameter matching and optimal design of dual-motor driven electric tractor. International Journal of Agricultural and Biological Engineering. 2019;12(1):33–41.

[23] Li T, Xie B, Li Z, et al. Design and optimization of a dual-input coupling powertrain system: A case study for electric tractors. Applied Sciences. 2020;10(5):1608.

[24] Wen C-k Zhang S-l, Xie B, et al. Design and verification innovative approach of dual-motor power coupling drive systems for electric tractors. Energy. 2022;247:123538.

[25] Guo H, Shangguan J, Tang J, et al. Receding horizon control strategy for an electric vehicle with dual-motor coupling system in consideration of stochastic vehicle mass. Plos one. 2018;13(10):e0205212. doi: 10.1371/journal.pone.0205212 30308000 PMC6181328

[26] Yang H, Sun Y, Xia C, et al. Research on Energy Management Strategy of Fuel Cell Electric Tractor Based on Multi-Algorithm Fusion and Optimization. Energies. 2022;15(17):6389.

[27] He R, Jimenez E, Savitski D, et al. Investigating the parameterization of dugoff tire model using experimental tire-ice data. SAE International Journal of Passenger Cars-Mechanical Systems. 2016;10(2016-01-8039):83–92.

[28] Li T, Xie B, Wang D, et al. Real- time adaptive energy management strategy for dual-motor-driven electric tractors. Transactions of the Chinese Society for Agricultural Machinery. 2020;51(S2):530–43.

[29] Deng X, Sun H, Lu Z, et al. Research on Dynamic Analysis and Experimental Study of the Distributed Drive Electric Tractor. Agriculture. 2023;13(1):40.

[30] Dou H, Wei H, Zhang Y, et al. Configuration Design and Optimal Energy Management for Coupled-Split Powertrain Tractor. Machines. 2022;10(12):1175.

[31] Xu L, Liu M, Zhou Z. Design of drive system for series hybrid electric tractor. Transactions of the Chinese Society of Agricultural Engineering (Transactions of the CSAE). 2014;30(9):11–8.

[32] Liu M, Li S, XU L, et al. Design and performance analysis of tractor bidirectional coupling electric drive system. Transactions of the Chinese Society for Agricultural Machinery. 2022.

