# Supplementary material for: Design and optimization of dual-motor electric tractor drive system based on driving cycles
Source: PLoS One. 2023 Jun 2;18(6):e0286378. doi: 10.1371/journal.pone.0286378 (PMC10237445; doi:10.1371/journal.pone.0286378)
Supplement: S1 File — (PDF) [file pone.0286378.s001.pdf]

In this paper, the POMBDC and the IO-CSRDM are used to distribute the torque of the traction motor and the PTO motor. Under plowing conditions, the torque distribution between the traction motor and the PTO motor is shown in the Fig 1 when the required speed and torque are given by using the POMBDC.

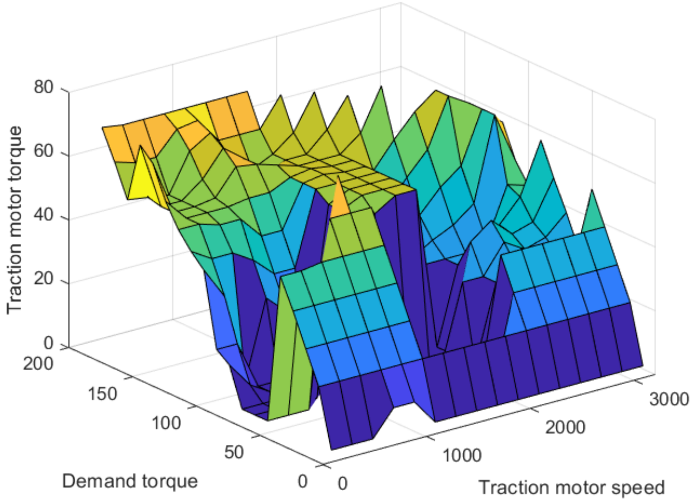

(a)

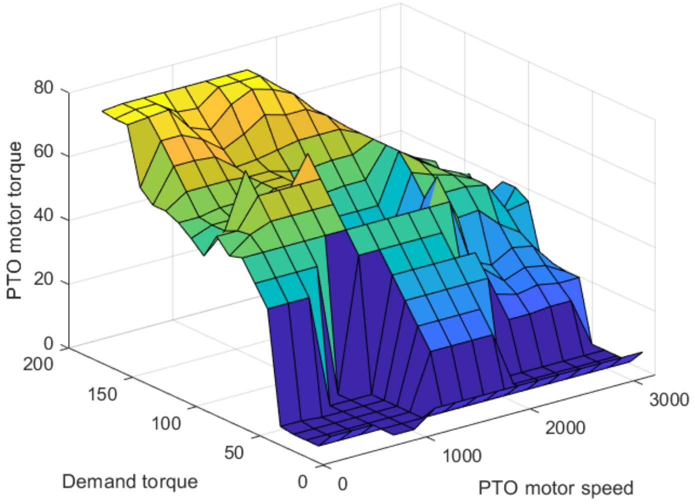

(b)

Fig 1. Torque distribution of two motors. (a) Traction motor. (b) PTO motor working point.

Under plowing conditions, the torque distribution between the traction motor and the PTO motor is shown in the Fig 2 when the required speed and torque are given by using the IO-CSRDM.

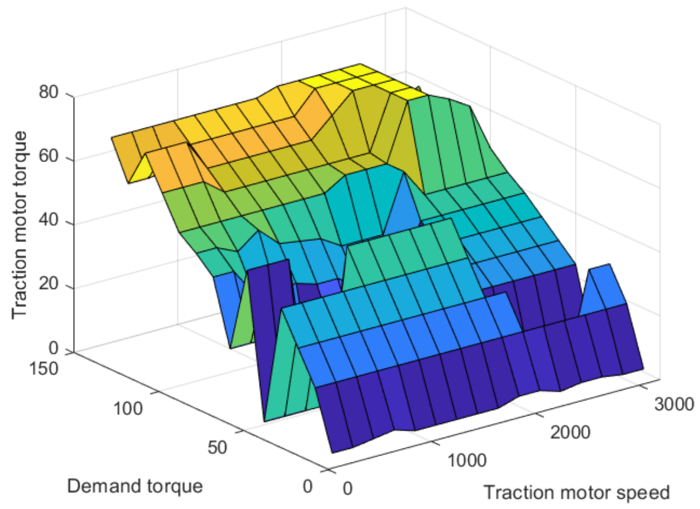

(a)

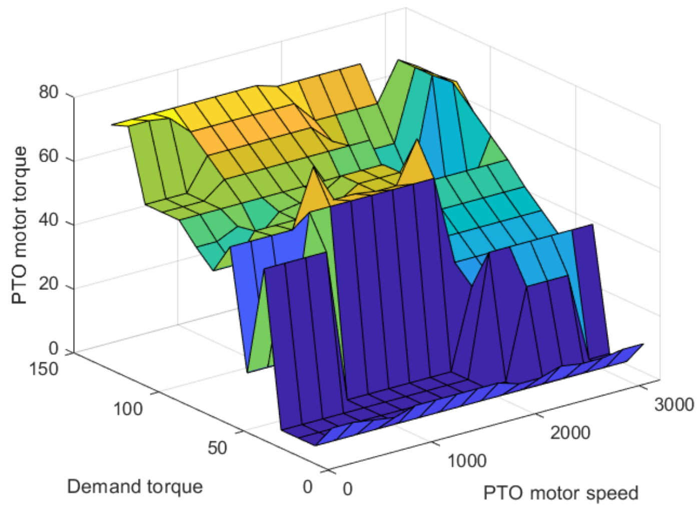

(b)

Fig 2. Torque distribution of two motors. (a) Traction motor. (b) PTO motor working point.

Under rotary tillage conditions, the torque distribution between the traction motor and the PTO motor is shown in the Fig 3 when the required speed and torque are given by using the POMBDC.

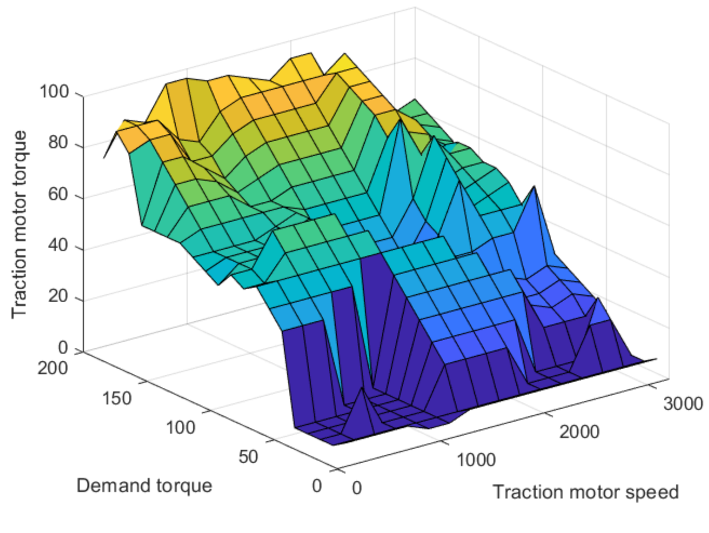

(a)

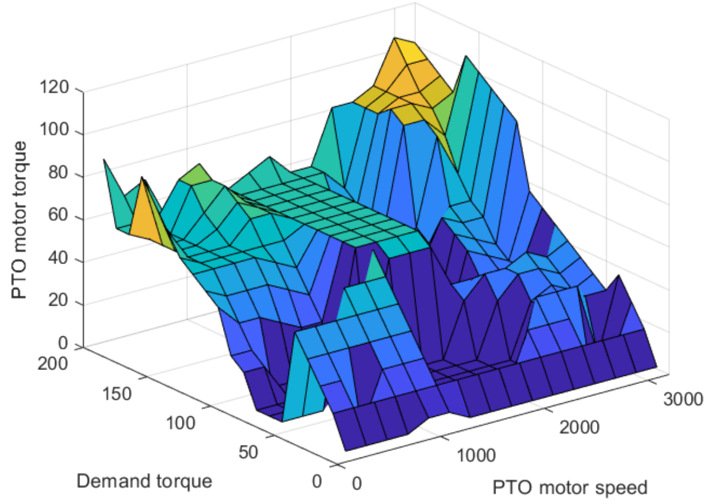

(b)

Fig 3. Torque distribution of two motors. (a) Traction motor. (b) PTO motor working point.

Under rotary tillage conditions, the torque distribution between the traction motor and the PTO motor is shown in the fig 3 when the required speed and torque are given by using the IO-CSRDM.

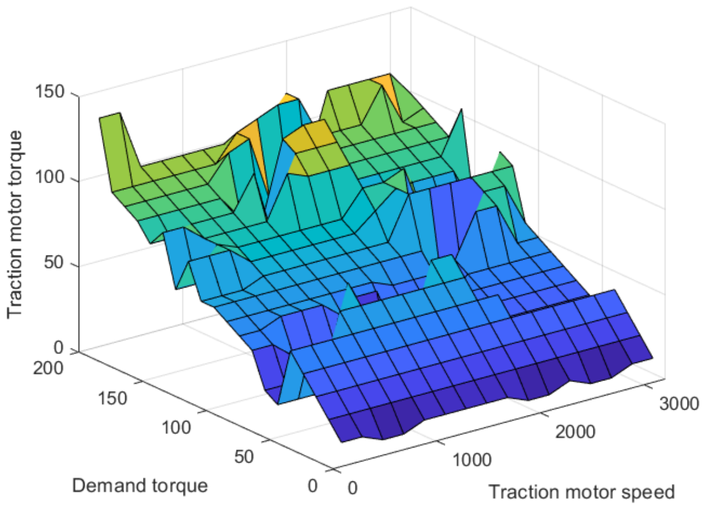

(a)

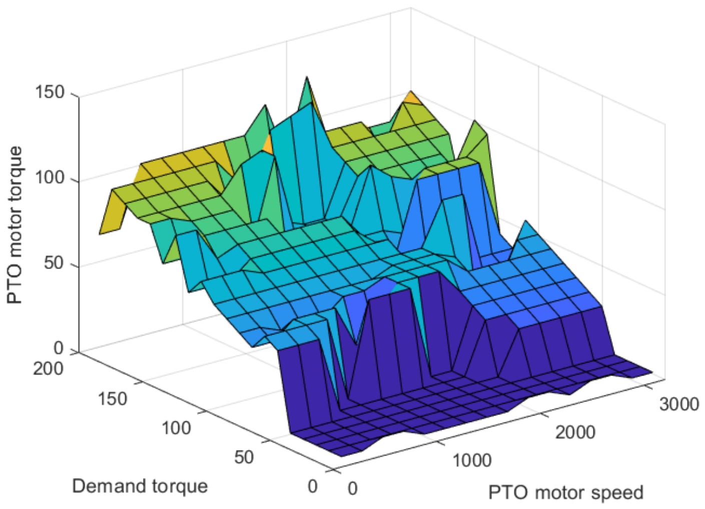

(b)

Fig 4. Torque distribution of two motors. (a) Traction motor. (b) PTO motor working point.
